# Metabolomic changes in *Cryptocaryon irritans* from *Larimichthys crocea* after exposure to copper plate

**DOI:** 10.3389/fcimb.2024.1424669

**Published:** 2024-06-28

**Authors:** Xiangyu Guo, Wenlian Huang, Yifan Xu, Quanjun Zhan, Peng Sun, Haojie Hu

**Affiliations:** ^1^ School of Marine Sciences, Ningbo University, Ningbo, China; ^2^ College of Animal Science, Zhejiang University, Hangzhou, China; ^3^ School of Oceanography, Shanghai Jiao Tong University, Shanghai, China

**Keywords:** Cryptocaryon irritans, copper plate, metabolome, tomonts, Larimichthys crocea

## Abstract

*Cryptocaryon irritans* is a highly detrimental parasite in mariculture, causing significant economic losses to the aquaculture industry of *Larimichthys crocea*. In recent years, copper and copper alloy materials have been used to kill parasites. In this study, the effect of copper plates on the tomont period of *C. irritans* was explored. The findings indicated that copper plates effectively eradicated tomonts, resulting in a hatching rate of 0. The metabolomic analysis revealed that a total of 2,663 differentially expressed metabolites (1,032 up-regulated and 1,631 down-regulated) were screened in the positive ion mode, and 2,199 differentially expressed metabolites (840 up-regulated and 1,359 down-regulated) were screened in the negative ion mode. L-arginine and L-aspartic acid could be used as potential biomarkers. Copper plate treatment affected 25 metabolic pathways in the tomont, most notably influencing histidine metabolism, retinol metabolism, the biosynthesis of phenylalanine, tyrosine, and tryptophan, as well as arginine and proline metabolism. It was shown that high concentrations of copper ions caused a certain degree of disruption to the metabolome of tomonts in *C. irritans*, thereby impacting their metabolic processes. Consequently, this disturbance ultimately leads to the rapid demise of tomonts upon exposure to copper plates. The metabolomic changes observed in this study elucidate the lethal impact of copper on *C. irritans* tomonts, providing valuable reference data for the prevention and control of *C. irritans* in aquaculture.

## 1 Introduction

*Cryptocaryon irritans* is a marine parasitic ciliate which is significantly harmful to marine fish farming industry. With the development of high density cage culture model of *Larimichthys crocea*, outbreaks of diseases caused by the *C. irritans* are frequent. The life history of *C. irritans* consists of four periods including trophont, protomont, tomont and theront (Watanabe et al., 2021). Under suitable temperature conditions, *C. irritans* can complete a life cycle in about 7 days, and a tomont can release 200-300 theront to reinfect *L.crocea.* Large-scale secondary infection is the main cause of marine fish mortality (Tang et al., 2022). Blocking one stage of the life history of *C. irritans* can block secondary infection and thus prevent mass death caused by the disease. The trophont stage is the parasitic stage of *C. irritans*, and the host fish is stimulated too much by taking control measures in this stage. The tomont period is the most prolonged period in the life history of *C. irritans*, during which tomonts will fall off from their host fish. Therefore, taking measures at this stage can prolong the operation time and reduce the harm to the host fish.

The way to prevent and control the diseases caused by this pathogen include physical, chemical and immunological methods (Hu et al., 2017; Gao et al., 2022; Lu et al., 2022), but they cannot be applied on a large scale due to the limitation of the open water cage culture mode. Physical methods include adjusting water temperature and ultraviolet (UV) irradiation. *C. irritans* is sensitive to temperature, and its growth and reproduction can be inhibited by raising the water temperature. However, in large-scale aquaculture environments, adjusting the water temperature is challenging and costly. Additionally, high temperatures can negatively impact fish, causing stress reactions or diseases. UV irradiation can effectively kill pathogens in the water, but its effective range is limited, making it difficult to cover large-scale aquaculture areas. Furthermore, UV irradiation equipment is expensive and requires regular maintenance (Diggles and Lester, 1996; Zhou et al., 2023). Chemical methods primarily involve the use of drugs or chemical agents (such as formalin, malachite green, and sodium chloride) to kill *C. irritans*. However, many chemical agents are toxic to fish and other aquatic organisms and can cause environmental pollution (Pironet and Jones, 2000; Zhong et al., 2023). Immunological methods include vaccination and the use of immune stimulants. These methods are complex to operate, costly, and individual immune responses can vary, leading to inconsistent control effect (Mo et al., 2022). Therefore, it is urgent to explore other effective prevention and control measures.

Copper, as a broad-spectrum antimicrobial material, has been widely used in anti-parasitic applications in recent years. Copper has many advantages as a means of controlling *C. irritans*. On the one hand, copper usually exists in the form of copper alloy cage, copper ion solution, etc., which does not require complicated operation steps or professional technical personnel. Copper, on the other hand, can be a long period of time continue to play a role of antibacterial, reduce the demand for frequent handling. In addition, copper do not cause environmental pollution as some chemicals. Therefore, copper is suitable for large-scale breeding application. It disrupts critical enzymatic activities in parasites by binding to thiol groups in proteins, thereby inhibiting their functions and leading to parasite mortality (Otludil and Ayaz, 2020; Li and Wu, 2021). The findings of certain studies suggest that mammals possess a specific mechanism for copper chelation, which effectively inhibits parasite growth without causing harm to the host organism (Grechnikova et al., 2024). This indicates the potential significance of copper in the defense mechanisms against parasites in various organisms. In aquaculture, treatment with 0.2 mg/L copper sulfate has been demonstrated to eradicate *Amyloodinium ocellatum* infestation on *Sardinella brasiliensis*, while without inducing any damage to the fish (Owatari et al., 2020). Some copper alloy coating was applied to tanks to block the life cycle by damaging the tomonts of *C. irritans*, and the reinfection rate and mortality of *Trachinotus ovatus and Epinephelus coioides* decreased significantly (Jiang et al., 2021). This study explores the effect of copper plates on tomonts of *C. irritans* based on metabolomics, enhancing our understanding of how copper disrupts parasites’ life processes, particularly its effects on crucial metabolic pathways. This research serves as a foundation for developing more precise prevention and control strategies, including targeted interventions in specific metabolic pathways, to optimize the efficacy of copper plate treatment.

## 2 Materials and methods

### 2.1 Sample collection

*Larimichthys crocea* infected with *C. irritans* were obtained from a farm located in Ningbo. Kept in the lab for 4 ~ 6 days until the tomonts fall off to the bottom. Collect and hatch the tomonts at 25 °C, theront that hatch from the tomonts infect *L. crocea* again. After obvious white spot appeared on *L. crocea*, transfer the fish to a clean bucket and collect the tomonts falling off at the bottom for subsequent experiment. Tomonts were collected every 24 h, and were washed with filtered seawater.

### 2.2 Statistics of hatching rate

The washed tomonts were divided into two groups: the seawater group (group CON, treated with filtered seawater) and the copper plate group (group EXP, exposed to copper plates in filtered seawater). After treatment, tomonts were placed in sterile seawater in 24 well plates and cultured at 27°C. The hatching number and hatching rate of tomonts were counted daily, then the tomonts were flash frozen in liquid nitrogen and subsequently stored at -80°C for metabolomics analysis.

### 2.3 Metabolite extraction

Accurately weigh a 100 mg mixtures of *C. irritans* tomonts from group CON and group EXP, add 1 ml pre-cooled tissue extract solution (75% 9:1 methanol: chloroform, 25% ddH_2_O) and grind them with tissue grinder. Then, ultrasound was carried out at room temperature for 30 min and placed on ice for 30 min. After centrifugation, the supernatant was concentrated. Acetonitrile solution was added to prepare 2-chlorophenylalanine solution to redissolve the sample, and then the sample was filtered for subsequent LC-MS detection.

### 2.4 Conditions for chromatography and mass spectrometry

Chromatographic separation was performed on a Thermo Ultimate 30,000 system equipped with an ACQUITY UPLC^®^ HSS T3 column maintained at 40 °C. Gradient elution of analytes was carried out with 0.1% formic acid in water (A) and 0.1% formic acid in acetonitrile (B) or 5 mM ammonium formate in water (C) and acetonitrile (D). Gradient elution procedure was performed: 0 ~ 1 min, 2% B/D; 1 ~ 9 min, 2%~50% B/D; 9 ~ 12 min, 50% ~ 98% B/D; 12 ~ 13.5 min, 98% B/D; 13.5 ~ 14 min, 98% ~ 2% B/D; 14 ~ 20 min, 2% B-positive model (14 ~ 17 min, 2% D-negative model). The ESI-MSn experiments were executed on the Thermo Q Exactive HF-X mass spectrometer. Full scan at resolution 60, 000, data dependent acquisition (DDA) MS/MS experiments were performed with HCD scan. Dynamic exclusion was implemented to remove some unnecessary information in MS/MS spectra.

### 2.5 Data processing and bioinformatics processing

The original data were converted into mzXML format by Proteowizard (v3.0.8789). The XCMS program package of R (v3.3.2) was used for peaks identification, peaks filtration and peaks alignment. Metabolites were obtained through the data matrix of mass to charge ratio, retention time, intensity and other information. After confirmation according to the accurate molecular weight of metabolites, the fragment information obtained from MS/MS was further matched. The annotation was based on Metlin (http://metlin.scripps.edu), MoNA (https://mona.fiehnlab.ucdavis.edu//), and self-built standard database. Autoscaling Mean-centering and scaled to unit variance (UV) was used for standardization. Orthogonal Projections to Latent Structures Discriminant Analysis (OPLS-DA) was used to determine the differences between treatment and control groups. The cross-validation of the model mainly refers to R^2^X, R^2^Y, Q^2^ and other parameters. The pheatmap program package in R was used to perform differential metabolite agglomerative hierarchical clustering on the dataset. The Kyoto Encyclopedia of Genes and Genomes (KEGG) and MetPA was used to query and annotate related metabolic pathways.

## 3 Results

### 3.1 Hatching rate of tomonts

**Figure 1:**
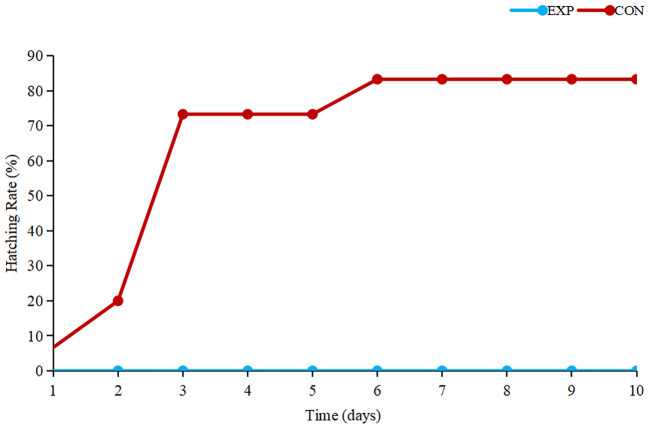
Hatching rate of tomonts.

According to the results (**Figure 1**), the control group of tomonts exhibited a maximum hatching rate within 5 days, from the initiation of the experiment until its completion, while the experimental group of tomonts failed to hatch. Hence, it is evident that copper plates have a pronounced killing effect on *C. irritans* tomonts.

### 3.2 OPLS-DA of C.irritans tomonts

**Figure 2:**
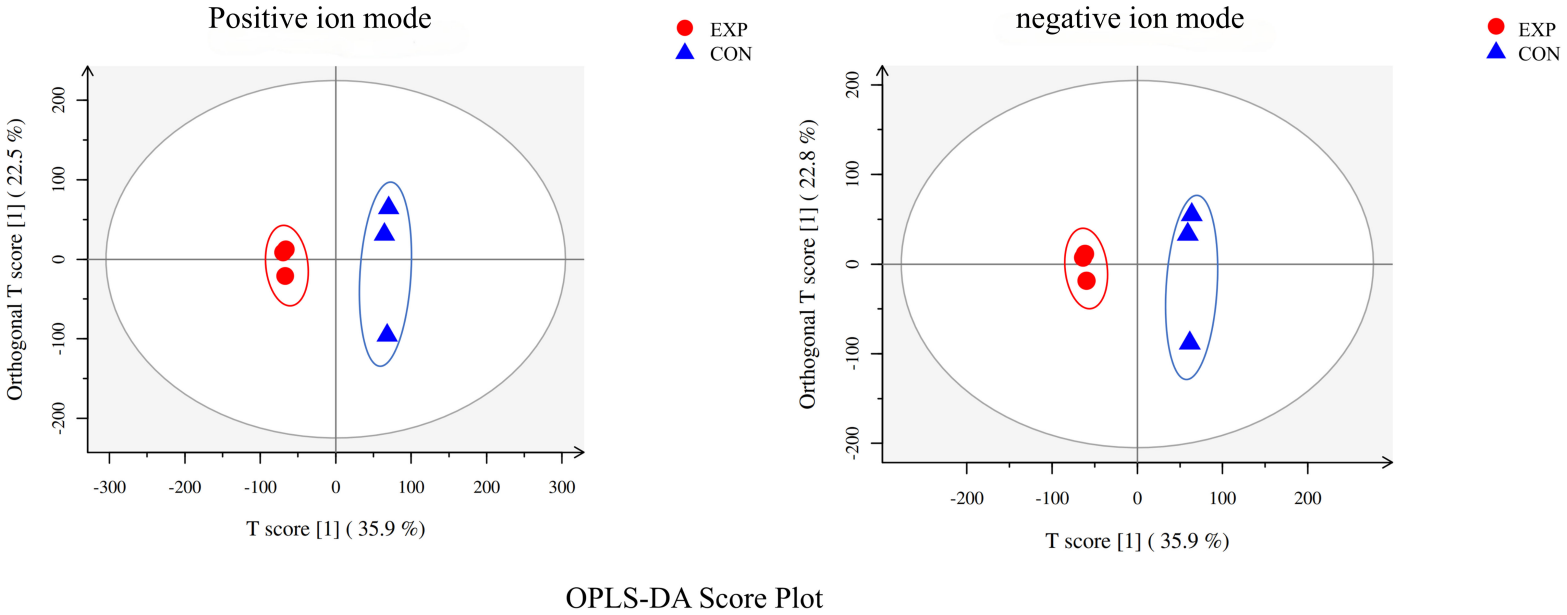
OPLS-DA analysis of positive and negative ion mode.

The metabolomic analysis determined the metabolic characteristics of tomonts after treated with copper plate, the ion peak had a stable retention time without drift in the chromatogram. The samples from the treatment and control groups were clustered separately in positive and negative ion mode, respectively. Orthogonal Projections to Latent Structures Discriminant Analysis (OPLS-DA) effectively extracted variation information and distinguished between the groups (**Figure 2**), indicating that copper plates altered the metabolome of *C. irritants* cysts. **Table 1** shows the multiple correlation coefficients of OPLS-DA (R^2^X = 0.584, R^2^Y = 0.999 in ion positive mode; R^2^X = 0.586, R^2^Y = 0.999 in negative ion mode) were all above 0.5, which signifies good repeatability in the test set. The cross-validation predictive ability (Q^2^ = 0.785 in positive ion mode and Q^2^ = 0.787 in negative ion mode) indicates the samples were normal, and no over fitting occurred in the test.

**Table 1:** Model validation parameters of OPLS-DA.

	pre	R^2^X	R^2^Y	Q^2^
positive ion mode	1 + 1+0	0.584	0.999	0.785
negative ion mode	1 + 1+0	0.586	0.999	0.787

Pre is principal component number; R^2^X is the interpretability of the model (for the x-variable dataset); R^2^Y is the interpretability of the model (for the y-variable dataset); Q^2^ is the degree of model predictability.

### 3.3 Screening of differential metabolites

**Figure 3:**
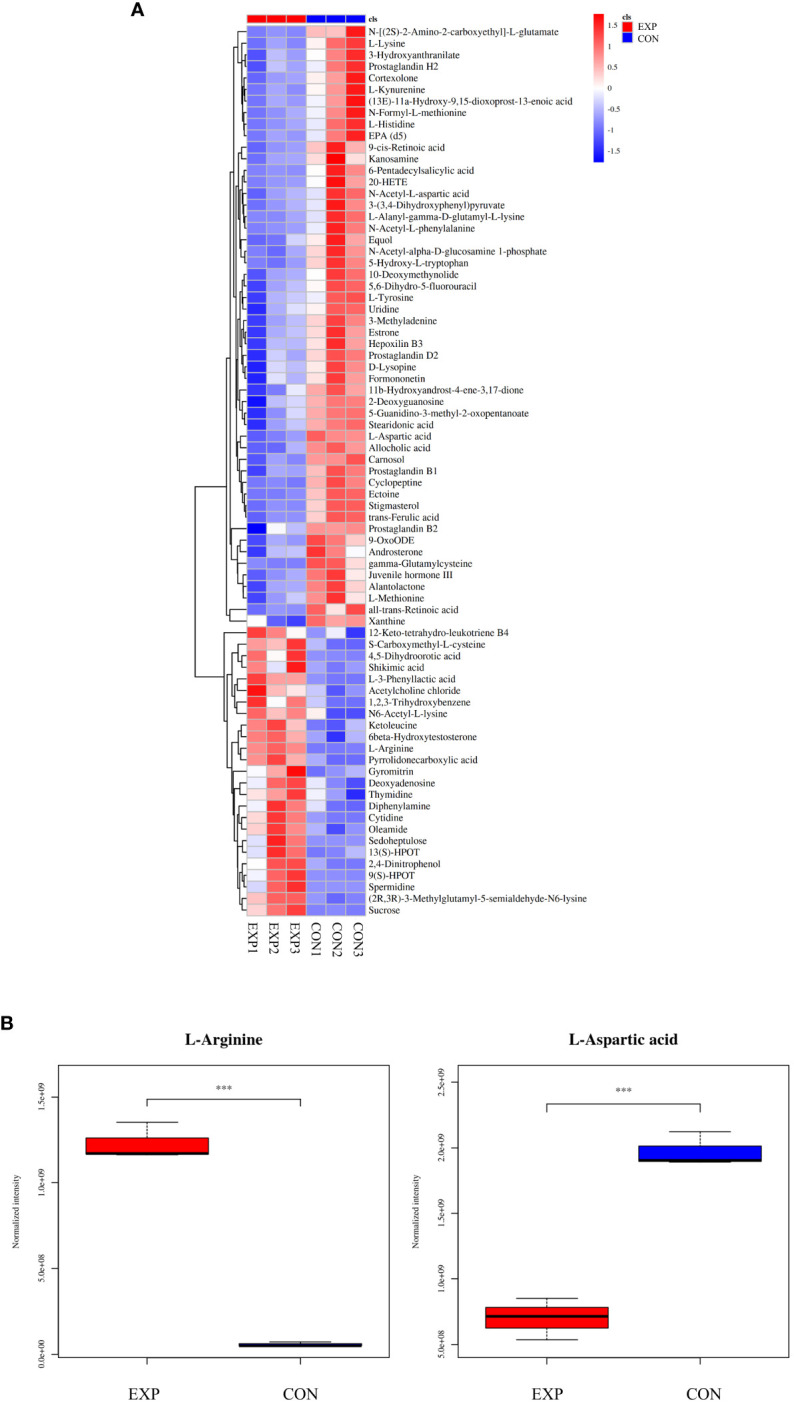
**(A)** Heat map of differential metabolites; **(B)** Box plot of differential metabolites. ***P < 0.001.

According to the condition of VIP ≥ 1, a total of 2,663 differential metabolites (1,032 up-regulated and 1,631 down-regulated) were screened in the positive ion mode, and 2,199 differential metabolites (840 up-regulated and 1,359 down-regulated) were screened in the negative ion mode (**Table 2**). Hierarchical clustering analysis was performed with the relative value of differential metabolites as the metabolic level. The heat map showed that 77 differential metabolites were significantly changed (25 up-regulated and 52 down-regulated) in the group EXP compared with the group CON (**Figure 3A**), among which L-arginine and L-aspartic acid changed significantly (*P* < 0.001), which could be used as potential biomarkers (**Figure 3B**).

**Table 2:** Statistical table of differential metabolite.

	Total	Up	Down
positive ion mode	2,663	1,032	1,631
negative ion mode	2,199	840	1,359

### 3.4 Changes in the metabolic pathways

**Figure 4:**
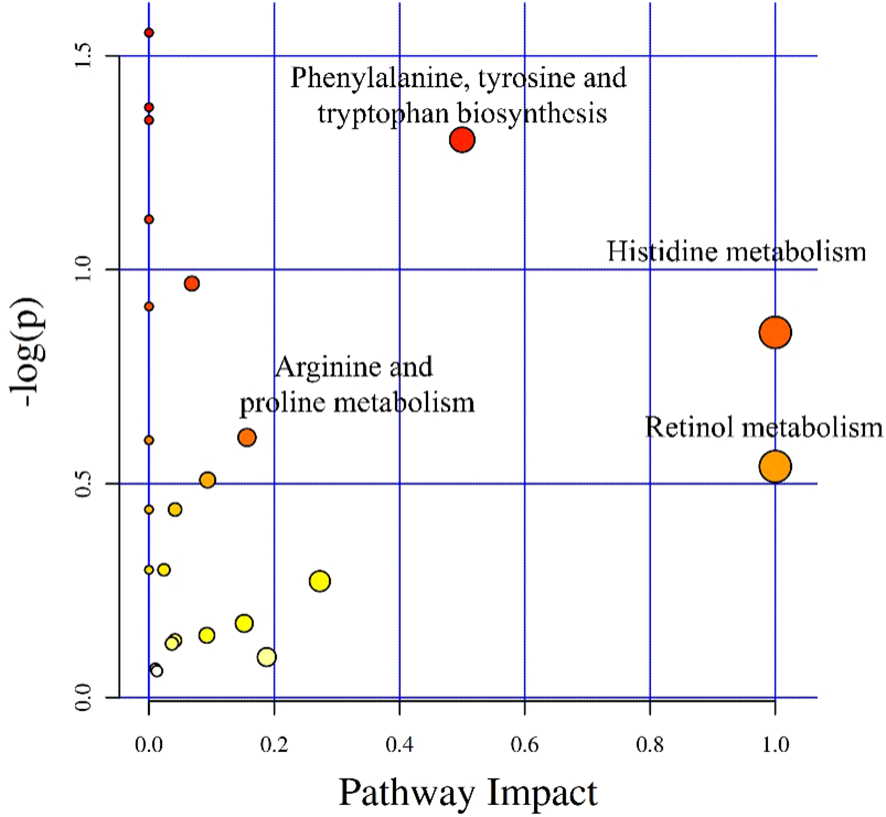
Differential metabolite pathway analysis. The X-axis represents the pathway impact, and the Y-axis represents -log(p).

After integrating KEGG and MetPA information, a total of 25 metabolic pathway of the tomonts were affected by the treatment of copper plate. The enrichment analysis of KEGG pathway showed that the p values of histidine metabolism, retinol metabolism, phenylalanine, tyrosine and tryptophan biosynthesis, as well as arginine and proline metabolism were low, which also had a great impact value on the pathway (**Figure 4**). Specifically, in the histidine metabolism pathway, the level of L-histidine was reduced. Retinol metabolism was affected by a decrease in all-trans-retinoic acid. In the biosynthesis pathways of phenylalanine, tyrosine, and tryptophan, there was a noted decrease in tyrosine. Conversely, in the arginine and proline metabolism, there was an increase in the levels of arginine and spermidine (**Table 3**). These changes suggest that the presence of copper plates disrupts the metabolic activities critical for the growth and development of *C. irritans* tomonts, potentially inhibiting their progression through the lifecycle and their ability to infect host organisms.

**Table 3:** KEGG pathways significantly enriched for metabolites.

Enrichment pathways	Different metabolites	Change
Histidine metabolism	L-Histidine	down
Retinol metabolism	All-trans-Retinoic acid	down
Phenylalanine, tyrosine and tryptophan biosynthesis	Tyrosine	down
Arginine and proline metabolism	Arginine Spermidine	up up

## 4 Discussion

White spot disease is considered to be the most harmful parasitic disease limiting the development of marine fish farming. Copper is a bactericidal material often used in aquaculture. It is very necessary to figure out the role of copper on tomonts. The studies have revealed that tomonts are capable of absorbing a substantial concentration of Cu^2+^ upon contact with a copper plate, which may cause membrane damage, subsequent oxidative stress, cellular apoptosis, and DNA degradation, ultimately resulting in organismal demise (Grass et al., 2011). In addition, When tomonts were treated with copper plates, the H2A, H2B, H3 and H4 proteins in the systemic lupus erythematosus pathway were significantly down-regulated, indicating that processes such as DNA replication were affected, blocking the division and hatching of theronts (Yin et al., 2019). In this study, after treatment with copper plates, the hatching rate of tomonts decreased to 0, indicating that copper plates could effectively kill tomonts by releasing copper ions after dissolution of copper plates in seawater, which penetrated through the tomonts and destroyed the growth mechanism of tomonts. The pivotal targets and mechanisms of copper-induced stress were examined through the observed changes in metabolomics.

An extensive array of differential metabolites were detected in the tomonts following copper treatment—2,663 in positive ion mode and 2,199 in negative ion mode. The wide variety of differential metabolites underscores copper’s capacity to penetrate and disrupt the fundamental biological processes of tomonts. Notably, L-arginine and L-aspartic acid were highly differentially expressed and may serve as potential biomarkers. The significant upregulation of L-arginine suggests changes in polyamine synthesis and nitric oxide pathways, which affected cell proliferation and survival (Castilho-Martins et al., 2015). In contrast, the considerable downregulation of L-aspartic acid could indicate an interruption in neurotransmitter synthesis, affecting energy metabolism and neural functions in tomonts (Hata, 1994). These pronounced alterations in amino acids imply that copper treatment interferes with nitrogen metabolic pathways, which are crucial for a range of cellular functions, including protein synthesis, signaling, and immune response regulation (James and Hibbs, 1990; Marchese et al., 2018; Bushra et al., 2023). In conclusion, the extensive changes in tomonts metabolism not only confirm copper’s efficacy in disrupting the life cycle of *C. irritans* but also emphasize the broad impact of copper-induced stress on cellular activities. Moreover, the plethora of differential metabolites identified post-copper treatment provides a valuable dataset for further investigation. By concentrating on the most significantly altered pathways, we can delineate the sequence of metabolic disruptions caused by copper and clarify the molecular mechanisms, ultimately contributing to targeted strategies for the prevention and control of *C. irritans* infections in aquaculture.

L-histidine has various physiological functions such as antioxidant, immunomodulatory and anti-inflammatory (Hara et al., 2013). In the context of copper-induced oxidative stress, it effectively mitigates intracellular toxicity by binding copper ions. For example, the homolog of LEA (Late Embryogenesis Abundant) found in *C. elegans* can combine with Cu^2+^ to form oligomers and polymers due to its histidine-rich nature. This binding reduces the intracellular Cu^2+^ concentration and alleviates the toxic effects of the metal on the cells (Gal et al., 2004; Liu et al., 2017). Given the antioxidant and metal-binding characteristics of histidine, its role in cellular protection is underscored by the observed decrease in L-histidine levels within the significantly impacted histidine metabolism pathway after tomonts were treated with copper plates. This decline may indicate an increased demand for histidine in tomonts, suggesting that histidine-rich proteins are being recruited to bind and neutralize excess Cu^2+^, counteracting the effects of increased copper levels.

Retinol metabolism is pivotal in regulating numerous essential physiological functions. Retinol is enzymatically converted into all-trans retinoic acid (ATRA), a critical metabolite of vitamin A, which influences various biological processes. These processes include embryonic development, cellular differentiation and communication, immune function, and vision (Hurst and Else, 2012; Thompson et al., 2019; Liang et al., 2021). ATRA contributes to the antioxidant role of vitamin A by modulating the expression of genes involved in antioxidant responses (Blaner et al., 2021). For instance, chondrocytes treated with retinoic acid exhibit increased activities of catalase, glutathione reductase, and superoxide dismutase, enhancing their protection against oxidative damage (Teixeira et al., 1996). In retinol metabolism, the content of all-trans-retinoic acid decreased, which may be due to the involvement of retinoic acid in the expression of antioxidant genes.

The uptake and metabolism of aromatic amino acids, including tryptophan, phenylalanine, and tyrosine, are vital for parasite survival. Studies using potent analogs have significantly disrupted the metabolism of these amino acids in parasites, suggesting that these analogs may directly target and inhibit these pathways (Cockram et al., 2020). Tyrosine, in particular, is not only a fundamental building block for protein synthesis but also a precursor to several bioactive molecules. It plays a crucial role in diverse biological functions, such as neurotransmission in the nervous system, hormone production in endocrine regulation, and energy yield in metabolism (Wheeler-Alm and Shapiro, 1992). Within the scope of this study, the reduction in tyrosine levels due to copper plate treatment might impair its role as a neurotransmitter precursor, thus potentially affecting signal transduction and behavioral responses in tomonts. This depletion may also interfere with the synthesis of thyroid hormones, subsequently impacting energy metabolism and growth in the parasite.

Studies have demonstrated that arginine plays a pivotal role in the biosynthesis of biological proteins and the generation of nitric oxide (NO). Additionally, it serves as the precursor for polyamine synthesis. Polyamines, such as spermidine, are intricately linked to cellular proliferation (Pegg and Casero, 2011). It plays a role in regulating cell growth and metabolism (Han et al., 2022). For instance, in Stevia rebaudiana subjected to salt stress, the application of exogenous spermidine has been shown to enhance the activity of antioxidant enzymes. This counters the reactive oxygen species produced internally due to environmental stress, and concurrently, it increases cell membrane stability (Bahari Saravi et al., 2022). Under abiotic stress, the level of spermidine in *Arabidopsis increases*, thereby enhancing tolerance (Alcázar et al., 2006) . In this experiment, the results showed that the content of arginine and spermidine increased significantly after tomonts contacted the copper plate, which can be guessed as a response to external stress. This allows the tomonts to maintain high antioxidant enzyme activity and maintain the redox state of the cells (Naz et al., 2021).

## 5 Conclusion

The treatment of *C. irritans* tomonts with copper plates in the present study significantly impacted their metabolome. L-arginine and L-aspartic acid can serve as biomarkers for tomonts in *C. irritans* under copper plate-induced stress. Annotation of differential metabolites revealed that copper plates primarily affected histidine metabolism, retinol metabolism, phenylalanine, tyrosine and tryptophan biosynthesis, as well as arginine and proline metabolism. These alterations in the metabolome indicate that high concentrations of Cu ions disrupt the normal metabolic processes and induce oxidative stress in tomonts, ultimately leading to their rapid demise following copper plate treatment. This study offers valuable insights into the potential mechanism of action of *C. irritans* tomonts under copper plate stress. Subsequent investigations could explore the effects of copper treatment on other life stages of *C. irritans*. There is also an urgent need to evaluate the long-term efficacy of copper therapy in controlling infections and to develop alternative therapies.

## Data availability statement

The raw data supporting the conclusions of this article will be made available by the authors, without undue reservation.

## Ethics statement

The animal study was approved by the Ethics Committee of Ningbo University. The study was conducted in accordance with the local legislation and institutional requirements.

## Author contributions

XG: Writing – original draft, Conceptualization, Software. WH: Writing – original draft, Methodology. YX: Writing – original draft, Software. QZ: Writing – original draft, Validation. PS: Writing – review & editing, Supervision. HH: Writing – review & editing, Supervision.
